# VA Nursing Home to Home Program: Mixed Methods Evaluation of an Innovative Two-Year Pilot

**DOI:** 10.1093/geroni/igaf122.4347

**Published:** 2025-12-31

**Authors:** Rachel Fix, Chelsea Manheim, Orna Intrator, Winifred Scott, Leah Haverhals

**Affiliations:** VA Health Services Research and Development, Aurora, Colorado, United States; VA, Aurora, Colorado, United States; James J Peters VA Medical Center, New York, New York, United States; VA, Menlo Park, California, United States; VA Eastern Colorado Healthcare System, Aurora, Colorado, United States

## Abstract

As the United States Veteran population ages, the Department of Veterans Affairs (VA) is expanding initiatives to ensure Veterans age at home. In 2023, the VA developed the Nursing Home to Home (NHTH) pilot program to assist Veterans living in VA-paid community nursing homes (CNH) to return home. This abstract describes cost savings and Veteran and caregiver satisfaction with NHTH. Cost analyses were conducted through analyzing 24 NHTH Veterans discharged from CNHs across three VA Medical Centers. Analyses were based on long-term services and supports (LTSS), assuming other costs did not change before/after NHTH discharge, during four, 30-day intervals before and after discharge. Average savings of LTSS was $5,496 per Veteran per month (equivalent to $65,952 per Veteran per year). Additionally, 67 satisfaction surveys were completed by Veterans and caregivers, at two points in time, post-CNH discharge. Qualitative interviews were conducted with 21 Veterans or their caregivers. We applied thematic analysis to analyze Veterans’ and caregivers’ satisfaction surveys and interview data. Results showed Veterans and caregivers having high satisfaction with NHTH. The NHTH dyadic teams of one nurse and one social worker acted as liaisons to Veterans receiving VA LTSS. LTSS received post-discharge facilitated Veterans’ safety once home. Evaluation results suggest NHTH to be a promising pilot program with a high likelihood of future success in ensuring Veterans placed in CNHs can transition safely back to the community. This is especially critical as VA costs for CNH care are projected to double by 2037.

